# Metabolic control of RNA methylation in rheumatoid arthritis: from synovial stress to pathogenic cellular adaptation

**DOI:** 10.3389/fimmu.2026.1865927

**Published:** 2026-07-17

**Authors:** Shu Li, Lei Wan, Jin Yang, Kun Wang, Xiaojun Zhang, Xiaochuang Liu

**Affiliations:** 1 The First Affiliated Hospital of Anhui University of Chinese Medicine, Hefei, China; 2 Anhui University of Chinese Medicine, Hefei, China

**Keywords:** cellular adaptation, cellular metabolism, epitranscriptomics, rheumatoid arthritis, RNA methylation, synovial microenvironment

## Abstract

Rheumatoid arthritis (RA) is a chronic autoimmune disorder characterized by persistent synovitis, invasive pannus formation, cartilage degradation, and bone erosion. Although metabolic reprogramming and epigenetic dysregulation are increasingly recognized as central features of rheumatoid arthritis, the mechanisms by which local metabolic stress is converted into durable pathogenic cellular states remain incompletely understood. Recent advances in epitranscriptomics suggest that dynamic RNA modifications, particularly RNA methylation, act as critical post-transcriptional regulators of immune and stromal cell adaptation. Local hypoxia, enhanced glycolytic flux, lactate accumulation, mitochondrial dysfunction, oxidative stress, and lipid metabolic imbalance collectively influence the expression, activity, substrate availability, and transcript selectivity of RNA methylation regulators. These metabolically conditioned RNA modification programs, including canonical N6-methyladenosine (m6A) and emerging non-m6A marks such as internal N7-methylguanosine (m7G), may help stabilize pathogenic phenotypes across multiple cell types. In fibroblast-like synoviocytes (FLS), RNA methylation sustains glycolytic fitness, invasive behavior, and resistance to apoptosis and ferroptosis. In macrophages, it reinforces inflammatory polarization and extracellular vesicle-mediated communication. In T cells and neutrophils, it contributes to Th17 skewing, defective autophagy, oxidative stress responses, and excessive neutrophil extracellular trap (NET) formation. We further discuss how RNA methylation integrates non-coding RNA networks, extracellular vesicle signaling, and regulated cell death pathways to maintain chronic synovial inflammation and tissue destruction. Finally, we highlight the translational implications of this metabolic–epitranscriptomic interface, including biomarker discovery, patient stratification, and microenvironment-informed therapeutic strategies. Targeting both metabolic stress and RNA methylation-dependent adaptation may provide new opportunities for precision-oriented intervention in rheumatoid arthritis.

## 1 Introduction

Rheumatoid arthritis (RA) is a chronic systemic autoimmune disease characterized by persistent synovial inflammation, pannus formation, cartilage degradation, and bone erosion, ultimately resulting in progressive joint destruction, functional impairment, and disability (1, 2). Although biologic agents and targeted synthetic disease-modifying antirheumatic drugs (DMARDs) have substantially improved clinical outcomes, a considerable proportion of patients still experience refractory disease, incomplete therapeutic responses, or continued structural progression (3, 4). These clinical challenges indicate that classical immune-centered models alone are insufficient to fully explain the chronicity, tissue destructiveness, and treatment resistance of RA.

An increasingly recognized concept in RA pathobiology is that persistent inflammation is tightly intertwined with sustained metabolic adaptation. The inflamed synovium constitutes a metabolically hostile microenvironment characterized by profound hypoxia, aberrant angiogenesis, intense nutrient competition, oxidative stress, and localized acidosis (5, 6). Within this niche, fibroblast-like synoviocytes (FLS), macrophages, and infiltrating lymphocytes undergo extensive metabolic remodeling, including a prominent shift toward glycolysis, lactate accumulation, mitochondrial dysfunction, and disruption of redox homeostasis (7, 8). Importantly, these metabolic alterations are not merely secondary consequences of inflammation. Instead, they actively support pathogenic proliferation, amplify inflammatory signaling, promote tissue invasion, and enhance resistance to stress-induced cell death.

In parallel, epitranscriptomics has emerged as an important layer of post-transcriptional regulation in inflammatory and autoimmune diseases. Among the numerous RNA modifications identified to date, RNA methylation is particularly notable for its dynamic and environment-responsive nature. N6-methyladenosine (m6A), the most abundant internal modification in eukaryotic messenger RNA, is reversibly regulated by methyltransferases, demethylases, and RNA-binding proteins, commonly referred to as “writers”, “erasers”, and “readers”, respectively. Through this regulatory machinery, m6A influences RNA splicing, stability, localization, nuclear export, and translational efficiency, The conceptual foundation of RNA methylation as a dynamic and reversible regulatory layer—pioneered by seminal discoveries in the early 2010s—has fundamentally reshaped our understanding of post-transcriptional control (9–13). Beyond m6A, an expanding repertoire of non-m6A RNA modifications is increasingly recognized as a critical component of cellular stress-adaptation programs in chronic inflammatory diseases.

To date, most studies on RNA methylation in RA have focused on its downstream roles in regulating inflammatory signaling pathways, shaping non-coding RNA networks, and modulating discrete pathogenic processes. However, a more fundamental and less explored perspective is that the metabolic microenvironment itself may directly instruct epitranscriptomic remodeling. In this view, hypoxia, acidosis, oxidative stress, mitochondrial dysfunction, and altered availability of metabolic substrates or cofactors collectively influence the expression, enzymatic activity, and transcript selectivity of RNA methylation regulators. This framework is particularly relevant to RA, because synovial stromal and immune cells are continuously exposed to fluctuating metabolic and inflammatory stressors.

Recent mechanistic studies increasingly support this paradigm. In RA synovial fibroblasts, RNA methylation regulators have been linked to enhanced glycolysis as well as resistance to apoptosis and ferroptosis. Moreover, metabolite-driven chromatin signaling, such as histone lactylation, has been shown to connect glycolytic end-products with RNA modification programs, supporting the concept that metabolites can actively shape the epitranscriptomic landscape. Related mechanisms in macrophages, T cells, and neutrophils further implicate RNA methylation in inflammatory polarization, defective autophagy, aberrant neutrophil extracellular trap formation, and overall disease activity. Together, these findings suggest that RNA methylation functions as a dynamic interface that translates metabolic stress into durable, cell-type-specific pathogenic adaptation.

Under sustained metabolic stress, pathogenic cells are not merely activated; they adapt. FLS acquire an aggressive, tumor-like phenotype with enhanced survival capacity, macrophages become locked into self-amplifying inflammatory circuits, and immune cell subsets develop maladaptive stress responses. In this Review, we discuss how these metabolic stressors reshape RNA methylation networks, stabilizing pathogenic cellular states and offering microenvironment-informed therapeutic strategies.

## 2 Epitranscriptomic rewiring in the RA synovial niche

In RA, the synovial niche is continuously exposed to severe hypoxia, enhanced glycolysis, lactate accumulation, oxidative stress, mitochondrial dysfunction, and altered lipid metabolism (14–17). Crucially, the relationship between metabolic stress and RNA methylation is not merely associative; rather, local metabolic perturbations actively dictate the epitranscriptomic landscape. For instance, profound synovial hypoxia does not simply coexist with altered methylation. Extrapolated from broader hypoxic disease models (such as carcinoma), HIF-1α stabilization may directly upregulate specific writers, such as METTL3, through transcriptional activation (18). Concurrently, the massive accumulation of lactate—a byproduct of synovial glycolytic rewiring—functions as a potent upstream epigenetic driver. Lactate induces targeted chromatin remodeling via histone lactylation (e.g., H3K18la), which directly promotes the transcription of the m7G writer METTL1 (19). Furthermore, severe oxidative stress (ROS) and lipid peroxidation in the synovial niche can alter the expression and stability of eraser proteins like ALKBH5 (20). Therefore, these metabolic stressors act as the primary architects of the RNA methylation machinery, defining its operational capacity and expression levels long before the downstream regulation of specific target transcripts occurs.

### 2.1 Hypoxia, glycolytic reprogramming, and lactate accumulation as initiating epitranscriptomic cues

A defining feature of the RA synovium is persistent hypoxia, which arises from synovial hyperplasia, dense inflammatory infiltration, aberrant angiogenesis, and an imbalance between local oxygen supply and metabolic demand (16). Under these conditions, resident FLS and infiltrating immune cells undergo a marked shift toward glycolysis. Although this metabolic switch enables rapid ATP generation under oxygen-limited conditions, it also promotes lactate accumulation and local acidification, thereby reinforcing the inflammatory and metabolically hostile synovial niche. Importantly, glycolytic remodeling is not simply a compensatory survival response; it actively supports RA-associated cell proliferation, migration, cytokine production, and stress tolerance (8).

Studies in chronic inflammatory settings further indicate that lactate accumulation can profoundly reshape immune cell metabolism, particularly in CD4^+^ T cells, and sustain pathogenic effector functions within inflamed tissues (21–23). These findings support the concept that hypoxia and glycolysis generate regulatory signals rather than inert metabolic byproducts. Consistent with this view, intermediate metabolites such as succinate can amplify inflammatory responses through HIF-1α-dependent pathways, further emphasizing that accumulated metabolites exert broad downstream regulatory effects (24).

System-level metabolomic analyses also demonstrate that RA is associated with extensive metabolic disturbance. Untargeted metabolomic studies have identified widespread alterations in lipid, amino acid, and nucleotide metabolism, as well as distinct immunometabolic signatures that distinguish patients with RA from healthy controls (25, 26).

Mechanistic evidence in RA-FLS has begun to directly connect metabolic remodeling with epitranscriptomic regulation. The USP5–METTL14–m6A–*GLUT1* axis has been reported to enhance glycolysis by stabilizing glucose transport-related signaling, thereby supporting the bioenergetic demands of activated synoviocytes (27). Among glycolysis-derived metabolites, lactate is particularly important. Once regarded primarily as a metabolic waste product, lactate is now recognized as a signaling molecule capable of influencing chromatin organization and gene regulatory programs (21, 28). In RA synovial fibroblasts, histone H3 lysine 18 lactylation has been linked to METTL1-mediated internal m7G modification of *NeuroD1*, thereby promoting resistance to ferroptosis (19).

This mechanism provides a disease-relevant example of a metabolic-to-epitranscriptomic regulatory route, indicating that lactate can act not only as a metabolic marker of inflammation, but also as an active regulator of RNA modification-dependent cellular adaptation.

### 2.2 Oxidative stress, mitochondrial dysfunction, and lipid perturbations as selective pressures

In addition to glycolytic remodeling, the RA synovial microenvironment is characterized by persistent oxidative stress, mitochondrial dysfunction, and lipid metabolic imbalance (26). Chronic inflammatory stimulation promotes excessive reactive oxygen species production, disrupts mitochondrial homeostasis, and perturbs redox balance. At the same time, altered lipid metabolism contributes to the generation of inflammatory mediators and influences cellular vulnerability to lipid peroxidation-associated forms of regulated cell death. This concept is consistent with the broader view that articular inflammation is closely linked to bioenergetic dysfunction. In RA and related inflammatory conditions, oxidative stress and mitochondrial abnormalities do more than cause nonspecific macromolecular damage; they reshape how cells prioritize survival, inflammatory output, and stress tolerance (29). Accordingly, mitochondria should be considered signaling hubs within the arthritic microenvironment, rather than simply impaired energy-producing organelles (30).

RNA methylation appears to be closely integrated into this adaptive process. In RA-FLS, METTL3 promotes ferroptosis resistance by enhancing the stability of *SLC7A11* mRNA through an m6A–IGF2BP2-dependent mechanism, thereby supporting the tumor-like phenotype of synovial fibroblasts (31). This pathway is particularly relevant because *SLC7A11* mediates cystine uptake, supports glutathione biosynthesis, and protects cells against oxidative and lipid peroxidation-induced damage. Similarly, METTL1-associated pathways suggest that RNA methylation may convert oxidative and lipid stress into pro-survival post-transcriptional programs (19, 31).

The functional importance of reader proteins further supports this model. IGF2BP3 has been identified as a pathogenic m6A reader in RA, and pharmacological targeting of IGF2BP3 by celastrol has been reported to attenuate inflammatory phenotypes (32).

Although the precise metabolic consequences of IGF2BP3 activity in RA require further clarification, these findings are consistent with the notion that RNA methylation readers selectively stabilize transcripts required for adaptation to inflammatory, oxidative, and metabolic stress.

### 2.3 Metabolic substrates, cofactors, and the biochemical permissiveness of RNA methylation

At the biochemical level, the catalytic activities of RNA methylation writers and erasers are intrinsically coupled to the intracellular availability of specific metabolic substrates and cofactors. For instance, RNA methyltransferases (writers), such as the METTL3/METTL14 complex, are heavily dependent on the universal methyl donor S-adenosylmethionine (SAM). Metabolic shifts within the inflamed joint that perturb one-carbon metabolism directly alter the SAM/SAH ratio; because the SAM/SAH stoichiometry serves as a direct dial controlling cellular methylation capacity, an altered ratio under metabolic stress actively modulates global m6A deposition on target mRNAs (33).

Conversely, RNA erasers such as FTO and ALKBH5 structurally belong to the Fe^2+^- and α-KG-dependent dioxygenase superfamily, meaning their catalytic efficacy strictly requires α-KG and Fe^2+^ as essential cofactors (34). In the hostile RA synovial microenvironment, mitochondrial dysfunction and altered metabolic flux can result in the depletion of α-KG or the concurrent accumulation of structural analogues like succinate, fumarate, or R-2HG. These accumulating metabolites competitively occupy the α-KG-binding pockets of erasers like FTO, effectively blocking their demethylation activity and leading to aberrant transcript hypermethylation that disrupts downstream mRNA stability tracks (35). Therefore, chronic metabolic disturbance in RA influences epitranscriptomic output by fundamentally altering the biochemical stoichiometry required for writer and eraser functionality.

Although direct evidence in RA remains limited, studies in related inflammatory diseases provide important proof of concept. In juvenile idiopathic arthritis, the expression of the m6A demethylases FTO and ALKBH5 is markedly reduced in monocytes isolated from inflammatory sites, suggesting that local inflammatory environments can suppress key epitranscriptomic erasers *in vivo* (36).

**Figure 1:**
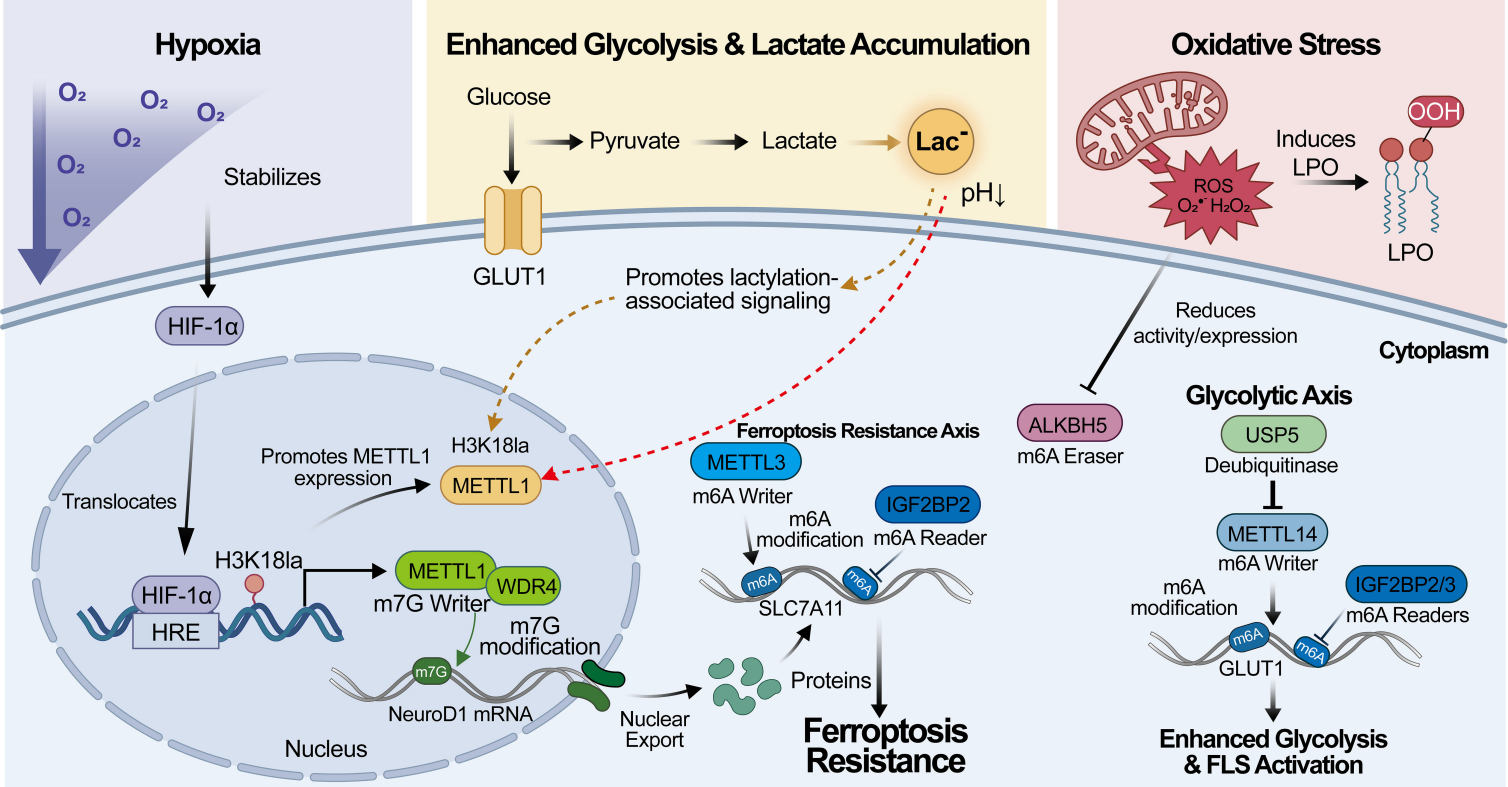
Metabolic-epitranscriptomic crosstalk contributes to pathogenic adaptation in RA -FLS. Synovial environmental stressors drive specific RNA modification rewiring events (including *SLC7A11*, *GLUT1*, and *NeuroD1* modification tracks) to lock fibroblast-like synoviocytes into an aggressive, apoptosis-resistant phenotype. ALKBH5, alkB homolog 5; GLUT1, glucose transporter 1; HIF-1α, hypoxia-inducible factor 1-alpha; HRE, hypoxia response element; IGF2BP2/3, insulin-like growth factor 2 mRNA-binding protein 2/3; LPO, lipid peroxidation; METTL1/3/14, methyltransferase-like 1/3/14; ROS, reactive oxygen species; SLC7A11, solute carrier family 7 member 11; USP5, ubiquitin-specific peptidase 5; WDR4, WD repeat domain 4.

The lactate–H3K18la–METTL1–*NeuroD1* axis described in RA synovial fibroblasts provides a disease-relevant example of multilayered metabolic control over RNA modification programs (19). More broadly, conceptual advances in epitranscriptomics emphasize that RNA modifications are dynamic outputs of environment-responsive regulatory systems, rather than static chemical decorations (37, 38). Within the RA synovium, the metabolic microenvironment may therefore function as a restrictive or permissive biochemical niche that determines not only which transcripts acquire RNA modifications, but also how these marks are interpreted through RNA stability, translation, localization, and decay (**Table 1**; **Figure 1**).

**Table 1:** Metabolic stressors in the rheumatoid arthritis synovial microenvironment and their epitranscriptomic consequences.

Microenvironmental stressor	Core metabolic impact	Epitranscriptomic consequence	Representative molecular nodes	Functional outcome	Ref.
Hypoxia	Glycolytic dependency & tissue acidification	Stress-responsive machinery reprogramming	HIF-related signaling; METTL3, METTL14, ALKBH5	Supports survival and persistence of pathogenic synovial cells	(17, 27, 39)
Enhanced glycolysis	Elevated glucose uptake & bioenergetic flux	Adaptive transcriptional program activation	USP5–METTL14–m6A–GLUT1 axis	Enhances FLS activation and metabolic fitness	(27)
Lactate accumulation	Dual metabolic-epigenetic signaling	Chromatin-to-RNA metabolic coupling	H3K18la–METTL1–NeuroD1–m7G cascade	Reinforces ferroptosis resistance and pathogenic adaptation	(19, 21, 28)
Oxidative stress	Redox imbalance & lipid peroxidation	Pro-survival epitranscriptomic selection	METTL3–IGF2BP2–SLC7A11 axis	Promotes resistance to ferroptotic stress	(29, 31)
Mitochondrial dysfunction	TCA cycle disruption & cofactor imbalance	Eraser inactivation via pocket inhibition	α-KG/Fe^2+^ dependency; FTO and ALKBH5 inactivation by 2-HG/succinate	Blocks transcript demethylation, driving aberrant mRNA hypermethylation and stability shifts	(16, 30, 34, 35)
One-carbon metabolic shift	Methionine cycle & SAM/SAH perturbation	Global m6A tuning via writer kinetics	SAM/SAH ratio; METTL3/METTL14 complex sensitivity	Drives transcript-specific stabilization or decay of inflammatory target genes	(33)
Lipid metabolic perturbation	Increases susceptibility to membrane damage	Epitranscriptomic-programmed cell death coupling	Ferroptosis circuitry	Contributes to chronic tissue injury	(31)

The inflamed synovium functions as an upstream biochemical niche that shapes RNA methylation dynamics and stabilizes disease-associated cellular states.

*2-HG, 2-hydroxyglutarate; α-KG, alpha-ketoglutarate; Fe^2+^, divalent iron; FLS, fibroblast-like synoviocytes; HIF, hypoxia-inducible factor; m6A, N6-methyladenosine; m7G, internal N7-methylguanosine; SAH, S-adenosylhomocysteine; SAM, S-adenosylmethionine; TCA, tricarboxylic acid.*

## 3 The RNA methylation landscape in RA: from canonical m6A to emerging non-m6A circuitry

If the synovial metabolic microenvironment establishes a restrictive chemical landscape in rheumatoid arthritis, RNA methylation serves as a key molecular interface that translates this pressure into functional post-transcriptional outputs. Among known epitranscriptomic marks, N6-methyladenosine (m6A) remains the most extensively characterized in RA. Dynamically regulated by writers, erasers, and readers, m6A controls multiple aspects of RNA metabolism, including splicing, nuclear export, stability, subcellular localization, and translation. Through these mechanisms, m6A enables cells to rapidly recalibrate gene expression in response to inflammatory and metabolic stress (34, 35).

Transcriptome-wide mapping studies have shown that m6A modifications are preferentially enriched near stop codons and within 3′ untranslated regions of mammalian mRNAs (9, 10). Subsequent identification of FTO and ALKBH5 as reversible RNA demethylases, WTAP as a regulatory component of the m6A methyltransferase complex, and m6A readers as determinants of mRNA stability and translational efficiency established m6A as a dynamic and reversible post-transcriptional regulatory system (11–13, 40–42).

However, the epitranscriptomic landscape of RA cannot be reduced to m6A alone. Findings from broader epitranscriptomic research highlight two important principles relevant to RA. First, the biological consequence of m6A is highly context-dependent and cannot be inferred solely from the expression level of a given writer, eraser, or reader (43–45). Second, cellular stress adaptation is also governed by non-m6A RNA modifications, including internal m7G, ac4C, and RNA editing, which can influence RNA stability, localization, translational efficiency, and intercellular signaling (46–48).

### 3.1 m6A-associated machineries and their dysregulation in RA

The m6A machinery in RA generally follows the classical tripartite model observed in other inflammatory and proliferative disorders, involving writers, erasers, and readers. Nevertheless, the pathogenic relevance of these factors in RA lies not simply in their altered expression, but in how they are selectively deployed to stabilize inflammatory, metabolic, and non-coding RNA-mediated regulatory programs.

METTL3, the catalytic core of the m6A writer complex, has been repeatedly implicated in RA pathogenesis. For example, METTL3-mediated m6A modification of the *circINTS4*/*miR-146b-3p* axis has been reported to accelerate RA progression (49). In macrophages, METTL3 promotes m6A-dependent maturation of *pri-miR-221*, thereby altering the M1/M2 polarization balance toward a pro-inflammatory state (50). Similarly, WTAP modulates macrophage polarization by regulating m6A modification of exosomal *circ-CBLB*, placing the m6A machinery at the center of pathological intercellular communication (51, 52).

m6A erasers exert similarly complex and cell-type-dependent functions. In FLS, ALKBH5 regulates apoptosis by modulating the maturation of *miR-181b-5p* through m6A demethylation (39). In neutrophils, reduced ALKBH5 expression correlates with clinical disease activity and is associated with impaired autophagy (20). In CD4^+^ T cells, m6A-linked *circFOXK2* alterations correlate with Th17 polarization and autophagic status (53).

Reader proteins provide an additional layer of selectivity by determining the fate of methylated transcripts. IGF2BP family proteins generally stabilize m6A-modified RNAs and enhance their translation (54). Consistent with this function, IGF2BP3 has been identified as a pro-pathogenic m6A reader in RA, and pharmacological targeting of IGF2BP3 by celastrol has been shown to alleviate inflammatory phenotypes (32). In contrast, YTHDF2 often functions as a decay-promoting reader. In RA, YTHDF2 has been reported to suppress synovial inflammation and bone injury by reducing the stability of *IL-6R* mRNA (55). Thus, m6A regulators in RA cannot be uniformly classified as pro-inflammatory or anti-inflammatory. Their functional output is determined by the modified transcript, the reader protein engaged, the cell type involved, and the metabolic state of the local microenvironment.

### 3.2 Beyond m6A: emerging evidence for non-m6A modifications in RA

As the field advances, non-m6A RNA modifications are increasingly recognized as important contributors to RA pathogenesis. Among these, internal m7G currently has the strongest disease-relevant mechanistic support. METTL1-mediated internal m7G methylation of Cathepsin B (*CTSB*) mRNA has been shown to promote synovial aggression, suggesting that m7G can facilitate translational programs required for the invasive behavior of synovial fibroblasts (56). When considered together with the lactate–H3K18la–METTL1–*NeuroD1* axis described in RA synovial fibroblasts, these findings position m7G as an emerging regulatory mechanism linking metabolic stress, translational control, and ferroptosis resistance (19, 25, 57).

To maintain pathophysiological rigor, it is crucial to distinguish between mechanisms directly validated in arthritic tissues and those inferred from alternative models. While internal m7G currently boasts direct disease-relevant validation in RA (e.g., the METTL1-*CTSB* axis in synovial aggression), other non-canonical marks remain conceptually hypothetical in this context. For instance, N4-acetylcytidine (ac4C), catalyzed primarily by NAT10, has been identified as a stability-enhancing modification in broader metabolic studies. It is important to note that direct evidence for ac4C in the RA joint is currently lacking; thus, the NAT10-ac4C axis serves primarily as a hypothesis-generating framework extrapolated from oncology and general inflammation (47, 57). Although direct evidence for ac4C in RA is still limited, studies in related inflammatory and metabolic settings suggest that NAT10-ac4C signaling may participate in macrophage activation, lipid metabolism, and autoimmune responses. Given the high metabolic and biosynthetic demands of the inflamed RA synovium, the NAT10-ac4C axis represents a plausible but underexplored epitranscriptomic mechanism in synovial macrophage and FLS reprogramming.

RNA editing further expands the scope of RNA-level regulation in RA. Adenosine-to-inosine editing mediated by ADAR enzymes can alter RNA structure, stability, coding potential, and immune recognition. In RA, ADAR1 has been reported to attenuate disease severity by regulating FLS-derived exosomal circFTO, indicating that RNA editing may shape the extracellular RNA landscape and influence intercellular communication within the inflamed joint (58, 59). Together, these observations support a broader epitranscriptomic model in which m6A, m7G, ac4C, and RNA editing may act either independently or cooperatively to regulate synovial adaptation.

### 3.3 The dynamic reversibility and context-dependency of RNA methylation networks

A defining feature of RNA methylation in RA is its reversibility and context dependency. The same regulator may produce divergent, or even opposing, biological effects depending on cellular lineage, target transcript availability, inflammatory stage, and metabolic state. For example, METTL3 contributes to inflammatory polarization in macrophages (49), whereas in FLS it is more closely associated with proliferation, ferroptosis resistance, and non-coding RNA-mediated regulation (31, 49). Likewise, ALKBH5 can regulate apoptosis in FLS, autophagy in neutrophils, and NETosis-associated lncRNA signaling in innate immune responses (20, 39). These observations indicate that RNA methylation networks should not be interpreted through a simple binary framework of “pathogenic” versus “protective”.

Instead, RNA methylation should be viewed as a regulatory grammar whose meaning is determined by transcript identity, reader availability, cellular context, and microenvironmental constraints. Within the RA synovium, metabolic stressors such as hypoxia, lactate accumulation, oxidative stress, and lipid peroxidation may alter not only the activity of RNA methylation enzymes, but also the repertoire of transcripts that are modified and the readers that interpret these marks. This context-sensitive behavior is particularly important for therapeutic development, because indiscriminate targeting of a globally expressed RNA methylation regulator may disrupt both pathogenic and protective pathways. A more precise understanding of cell-type-specific RNA methylation circuits will therefore be essential for translating epitranscriptomic discoveries into clinically meaningful interventions (**Table 2**).

**Table 2:** Major RNA methylation regulators in rheumatoid arthritis and their cell-type-specific roles.

RNA modification class	Key regulator(s)	Main cell type(s)	Molecular action	Biological effect	Disease implication	Ref.
m6A writer	METTL3	FLS, macrophages	Target coding/ncRNA methylation	Inflammation, proliferation, & stress tolerance	Generally pro-pathogenic	(31, 49, 50)
m6A writer	METTL14	FLS	m6A-dependent metabolic/translational control	Promotes glycolytic adaptation	Maintains synoviocyte fitness	(27)
m6A eraser	ALKBH5	FLS, neutrophils	m6A demethylation; miRNA precursor regulation	Modulation of apoptosis and cell autophagy	Context-dependent	(20, 39)
m6A reader	IGF2BP2/IGF2BP3	FLS and inflammatory cells	Transcript stabilization & translation promotion	Pathogenic transcriptome maintenance	Potential therapeutic targets	(31, 32)
m6A reader	YTHDF2	FLS	Promotes decay of inflammatory mRNAs	Restrains synovial inflammation	Generally protective	(55)
m6A-associated regulator	WTAP	Macrophages	Coordinates methyltransferase complex activity and exosomal RNA regulation	Modulates macrophage polarization	Amplifies inflammatory signaling	(51)
m7G writer	METTL1	FLS	Catalyzes internal m7G modification	Promotes invasive growth and ferroptosis resistance	Emerging pro-pathogenic factor	(19, 56)
RNA editor	ADAR1	FLS and exosomal pathways	Shapes RNA editing-dependent communication	Attenuates disease severity in reported models	Potentially protective	(58)
ncRNA-linked circuitry	circINTS4, circCBLB, circFOXK2, lncRNAs	FLS, macrophages, T cells	Mediates intercellular information transfer	Reinforces inflammatory persistence	Important disease amplifiers	(49, 51–53)

Functional effects are highly context-dependent and may vary across cellular compartments and disease stages.

*ADAR1, adenosine deaminase RNA specific 1; circ, circular RNA; CTSB, cathepsin B; IL-6R, interleukin-6 receptor; lncRNA, long non-coding RNA; miRNA, microRNA; ncRNA, non-coding RNA; WTAP, WT1-associated protein.*

## 4 Cellular state adaptation as the core functional output of metabolically sculpted RNA methylation in RA

### 4.1 Fibroblast-like synoviocytes: invasive growth, metabolic persistence, and evasion of cell death

Among RA-relevant cell populations, fibroblast-like synoviocytes provide one of the clearest examples of how metabolically conditioned RNA methylation can stabilize a pathogenic cellular state. Despite continuous exposure to hypoxia, acidosis, nutrient stress, and oxidative injury, RA-FLS do not simply undergo exhaustion or elimination. Instead, they acquire a tumor-like phenotype characterized by excessive proliferation, invasive behavior, resistance to apoptosis, and the capacity to directly mediate cartilage and bone destruction (6, 7).

A central component of this adaptive phenotype is the maintenance of enhanced glycolysis. The USP5–METTL14–m6A–*GLUT1* axis promotes glucose uptake and glycolytic flux in RA-FLS, thereby providing the bioenergetic support required for synoviocyte activation and persistence (27). This metabolic adaptation is closely linked to cell survival programs. ALKBH5 regulates apoptosis in RA-FLS through m6A-dependent modulation of *miR-181b-5p* maturation (39), whereas YTHDF2 acts as a restraining mechanism by destabilizing *IL-6R* mRNA and attenuating synovial inflammation and bone injury (55).

Resistance to ferroptosis represents another defining feature of RA-FLS adaptation. METTL3 enhances the stability of *SLC7A11* mRNA through an m6A–IGF2BP2-dependent mechanism (31), thereby strengthening antioxidant defense and reducing susceptibility to lipid peroxidation-induced cell death (31). Non-m6A pathways further reinforce this stress-resistant phenotype. METTL1-mediated internal m7G methylation of *CTSB* mRNA promotes synovial invasiveness (56), while the lactate-driven H3K18la–METTL1–*NeuroD1* pathway enhances ferroptosis resistance in RA synovial fibroblasts (19). In addition, epitranscriptomic regulation of non-coding RNA networks, including the METTL3-regulated *circINTS4*/*miR-146b-3p* axis (49) and ADAR1-dependent modulation of FLS-derived exosomal *circFTO* (58), suggests that RNA-level regulation not only shapes intrinsic FLS behavior but also influences the extracellular signaling landscape of the inflamed synovium.

Together, these studies indicate that RNA methylation helps RA-FLS integrate metabolic pressure with survival, invasion, and intercellular communication programs. This integration may explain, at least in part, why activated FLS persist within the synovium and continue to drive tissue destruction even when upstream inflammatory signals are therapeutically suppressed.

### 4.2 Macrophages: inflammatory polarization, stress adaptation, and niche amplification

Macrophages are central amplifiers of synovial inflammation, producing cytokines, chemokines, proteases, and metabolic mediators that sustain the inflammatory niche. RNA methylation regulates macrophage activation by shaping both intracellular inflammatory programs and extracellular communication pathways. METTL3-mediated m6A modification promotes *pri-miR-221* maturation, thereby altering the M1/M2 polarization balance toward a pro-inflammatory phenotype in RA (50). In addition, the *piENOX2*–ALKBH5–*Itga4* axis links m6A demethylation to disease-promoting signaling, suggesting that eraser-dependent regulation may also contribute to macrophage-associated RA progression (60).

Intercellular communication further amplifies macrophage-driven inflammation. WTAP modulates macrophage polarization by regulating m6A modification of exosomal *circ-CBLB* (51), while related studies on *circ-CBLB* and ETS-1 highlight the potential clinical relevance of this RNA-centered signaling axis (52). Reader-associated mechanisms also contribute to macrophage and inflammatory cell adaptation. For example, pharmacological targeting of IGF2BP3 by celastrol has been reported to attenuate RA severity, supporting the therapeutic relevance of reader-mediated transcript stabilization (32).

These findings are consistent with the broader framework of immunometabolism, in which macrophage inflammatory states are tightly coupled to metabolic remodeling (43, 61). Within the RA synovium, hypoxia, lactate accumulation, mitochondrial stress, and altered redox balance may therefore provide the environmental conditions that shape macrophage RNA methylation programs. Through this mechanism, macrophages may become locked into self-reinforcing inflammatory circuits that continuously amplify synovial pathology.

### 4.3 T cells and neutrophils: maladaptive immune responses under chronic metabolic pressure

Although FLS and macrophages currently dominate the mechanistic literature on RNA methylation in RA, adaptive and innate immune cells also undergo substantial epitranscriptomic remodeling under chronic metabolic pressure. T cells are particularly sensitive to nutrient availability, lactate accumulation, and mitochondrial fitness (62). Lactate-rich inflammatory environments can impair or redirect T cell bioenergetics and functional persistence within inflamed tissues (63, 64). In parallel, m6A-dependent post-transcriptional regulation is known to be critical for T cell homeostasis and cytokine responsiveness, suggesting that RNA methylation may shape adaptive immune balance in metabolically stressed inflammatory niches (65).

In RA, m6A-associated *circFOXK2* in CD4^+^ T cells correlates with Th17 frequencies and autophagic status (53). This relationship is notable because Th17-mediated immunity contributes to synovial inflammation and structural damage (53, 65). Although the mechanistic directionality between *circFOXK2* methylation, autophagy, and Th17 skewing requires further investigation, current evidence suggests that RNA methylation-linked circRNA networks may participate in the maladaptive persistence of autoreactive or pro-inflammatory T cell states.

Neutrophils represent another important immune population in which RNA methylation intersects with metabolic and oxidative stress. Reduced ALKBH5 expression in neutrophils has been associated with RA disease activity and dysregulated autophagy (20). This finding is particularly relevant in the context of neutrophil extracellular trap formation, a process tightly linked to oxidative stress and inflammatory tissue damage (**Table 3**).

**Table 3:** Cell type-specific adaptive programs shaped by metabolically conditioned RNA methylation in rheumatoid arthritis.

Cell type	Dominant metabolic phenotype	Key epitranscriptomic events	Adaptive pathogenic phenotype	Contribution to disease progression	Ref.
FLS	High glycolytic flux, lactate-rich, redox-stressed	SLC7A11 m6A stabilization; invasive transcript m7G regulation; circRNA signaling	Hyper-proliferation, invasion, & cell death resistance	Direct tissue destruction and pannus expansion	(19, 27, 31, 39, 49)
Macrophages	Immunometabolic reprogramming toward inflammatory activation	pri-miR-221 maturation; exosomal circRNA signaling	M1 polarization & inflammatory amplification	Sustains cytokine-rich synovial niche	(50, 51, 60)
CD4^+^ T & B cells	Lactate stress & proposed methionine shift (Extrapolated)	m6A-circFOXK2 tracks; EZH2-stimulated METTL3 activation	Th17 skewing, autophagy imbalance, & B-cell hyper-activation	Autoimmune effector response & autoantibody production	(53, 66)
Neutrophils, DCs, Mast cells	Oxidative stress & predicted glycolytic/hypoxic activation (Predicted)	Reduced ALKBH5 trails; METTL3/ALKBH5 dysregulation; YTHDC1/CLP1 rewiring	Dysregulated autophagy, hyper-immunogenic maturation, & lowered degranulation threshold	Local damage spread & autoreactive T-cell priming	(20, 69, 70)
Osteoclasts & Chondrocytes	Hypothetical HK1 glycolytic flux & predicted metabolic homeostasis defect (Extrapolated)	FTO-mediated HK1/USP14 m6A demethylation; METTL3-mediated ATG7 m6A hypermethylation	Accelerated osteoclastogenesis, cell fusion, & chondrocyte autophagy arrest	Subchondral bone erosion & extracellular matrix degradation	(67, 68)
EV network	Microenvironment-shaped cargo selection	m6A and RNA editing-dependent RNA sorting	Intercellular dissemination of pathogenic information	Reinforces chronic inflammatory loops	(51, 52, 58)

The same epitranscriptomic factor can exert distinct effects depending on lineage identity, activation state, and metabolic context.

*ATG7, autophagy-related 7; CLP1, CLP1 cleavage and polyadenylation factor; DC, dendritic cell; EV, extracellular vesicle; EZH2, enhancer of zeste homolog 2; HK1, hexokinase 1; M1, M1 macrophage polarization; Th17, T helper 17 cells; USP14, ubiquitin-specific peptidase 14; YTHDC1, YTH domain-containing protein 1.*

Collectively, these observations suggest that RNA methylation contributes to maladaptive immune remodeling in RA by regulating autophagy, effector differentiation, oxidative stress responses, and NETosis. However, compared with FLS, the causal mechanisms in T cells and neutrophils remain less fully defined and warrant further study using cell-type-specific and spatially resolved approaches.

### 4.4 B cells and pathogenic autoantibody production

Beyond T cell-mediated immunity, the loss of B cell tolerance and the subsequent production of pathogenic autoantibodies represent a core immunopathological hallmark of chronic joint inflammation. Although direct evidence within the arthritic lymphoid niche remains scarce, insights extrapolated from sister systemic autoimmune conditions suggest that RNA m6A modification acts as a potential gatekeeper of B-cell activation. For instance, in primary Sjögren’s syndrome, the histone methyltransferase EZH2 has been shown to promote B-cell autoimmunity by driving METTL3-mediated m6A modifications (66).

### 4.5 Osteoclasts and mediating bone erosion

Irreversible bone erosion in RA is primarily executed by hyper-activated osteoclasts. The differentiation of precursor cells into mature, bone-resorbing osteoclasts is governed by the RANKL pathway. While not yet verified directly in RA synovial tissue, mechanistic evidence derived from an apical periodontitis rat model suggests that this bone-resorbing axis may be modulated by m6A erasers (67). Specifically, the m6A demethylase FTO plays a pivotal role in promoting osteoclastogenesis and subsequent bone resorption. FTO-mediated eraser activity dynamically reprograms the epitranscriptomic status of the metabolic and signaling hub involving hexokinase 1 (*HK1*) and ubiquitin-specific peptidase 14 (*USP14*). This FTO-associated *HK1*/*USP14* axis substantially stabilizes and amplifies RANK signaling outputs, thereby escalating downstream osteoclast fusion and bone-erosive activity. This demonstrates that targeted RNA demethylation serves as a metabolic-signaling rheostat fueling subchondral bone destruction.

### 4.6 Chondrocytes and cartilage degradation

Articular cartilage loss, mediated by specialized chondrocytes undergoing phenotypic shifts and apoptosis, leads directly to joint space narrowing and permanent disability. Extrapolating from data in knee osteoarthritis models, chondrocytes under chronic inflammatory stress are proposed to exhibit epitranscriptomic reprogramming that disrupts joint homeostasis (68). The m6A writer METTL3 functions as a critical instigator of this degenerative cascade by specifically targeting autophagy-related 7 (*ATG7*) transcripts. METTL3-mediated m6A hypermethylation of *ATG7* mRNA suppresses its stability and translation, thereby severely impairing protective chondrocyte autophagy. Deprived of this essential survival mechanism, chondrocytes undergo accelerated apoptosis and concurrently upregulate matrix metalloproteinases (MMPs), culminating in the enzymatic digestion of the extracellular matrix and irreversible cartilage breakdown.

### 4.7 Dendritic cells and aberrant antigen presentation

Dendritic cells (DCs) serve as essential immunological bridges linking innate microenvironmental cues to adaptive autoimmune responses within the arthritic synovium. Accumulating evidence from sister systemic autoimmune diseases highlights RNA methylation as a pivotal post-transcriptional checkpoint gating DC behavior. Specifically, multi-cohort transcriptomic analysis in primary Sjogren’s syndrome has demonstrated that key m6A regulators, including METTL3 and the eraser ALKBH5, are comprehensively linked with abnormal dendritic cell infiltration and the activation of downstream autophagy pathways (69). By extending this paradigm from primary Sjögren’s syndrome, dysregulated m6A deposition is hypothesized to prevent the timely decay of activation-related transcripts in synovial DCs. This speculative epitranscriptomic stabilization could lock DCs into a hyper-immunogenic status, excessively priming autoreactive T cells and promoting downstream Th1/Th17 differentiation.

### 4.8 Mast cells and inflammatory degranulation

Mast cells are critical resident granular cells in the synovial sublining that actively drive early inflammation and tissue remodeling. Advanced computational profiling of RA synovial tissue has now formally bridged the gap between RNA modification and mast cell dynamics. Recent bioinformatic modeling utilizing multiple Gene Expression Omnibus (GEO) datasets identified core RNA modification regulators (such as the m6A reader *YTHDC1* and the RNA-processing factor *CLP1*) as core diagnostic hubs in RA, with their expression patterns showing a striking negative correlation with mast cell infiltration (70). This epitranscriptomic rewiring under synovial metabolic stress suggests that disrupted RNA processing networks lower the operational threshold for mast cell activation, tilting the synovial niche toward accelerated degranulation, hyper-secretion of tissue-destructive mediators, and subsequent joint destruction.

## 5 Pathological persistence: how RNA methylation integrates intercellular communication and cell fate programs

### 5.1 Dissemination of pathogenic information via non-coding RNAs and extracellular vesicles

One of the defining mechanisms underlying disease persistence in RA is the propagation of pathogenic information across cellular networks. This process is mediated in part by non-coding RNAs and extracellular vesicles. RNA methylation regulates multiple steps in this process, including the biogenesis, stability, localization, and functional activity of circular RNAs, microRNAs, and long non-coding RNAs, as well as the selective packaging of RNA cargo into extracellular vesicles released within the synovial microenvironment (59, 71).

A representative example is the METTL3-dependent regulation of the *circINTS4*/*miR-146b-3p* axis, which has been linked to RA progression (49). This mechanism suggests that m6A modification can influence disease activity by modulating circRNA-centered competing endogenous RNA networks. In macrophages, WTAP-mediated m6A modification of exosomal *circ-CBLB* regulates macrophage polarization, indicating that the m6A machinery can also shape extracellular vesicle-mediated communication between immune and stromal compartments (51). Related evidence involving *circ-CBLB* and ETS-1 further supports the potential relevance of this RNA regulatory axis as both a biomarker and therapeutic target in RA (52).

RNA methylation-associated circular RNA networks also participate in adaptive immune regulation. In CD4^+^ T cells, m6A-associated *circFOXK2* correlates with Th17 frequencies and autophagic status, suggesting that epitranscriptomic modulation of circRNAs may influence T cell differentiation and stress adaptation (53). These findings are particularly relevant because Th17-skewed responses contribute to persistent synovial inflammation and joint damage.

Extracellular vesicle-associated pathways extend RNA methylation-mediated regulation beyond the intracellular space. However, much of the detailed molecular machinery dictating epitranscriptomic EV cargo sorting is currently inferred from tumor biology. Direct evidence in RA is restricted to initial reports, such as ADAR1 attenuating RA severity by modulating FLS-derived exosomal *circFTO*. Therefore, the broader EV-epitranscriptomic sorting networks in the synovium remain to be definitively mapped (58). In addition, long non-coding RNAs such as *TGFB2-OT1* may function as competing endogenous RNAs and potential diagnostic biomarkers, further supporting the concept that RNA-centered intercellular signaling contributes to RA chronicity (72).

**Figure 2:**
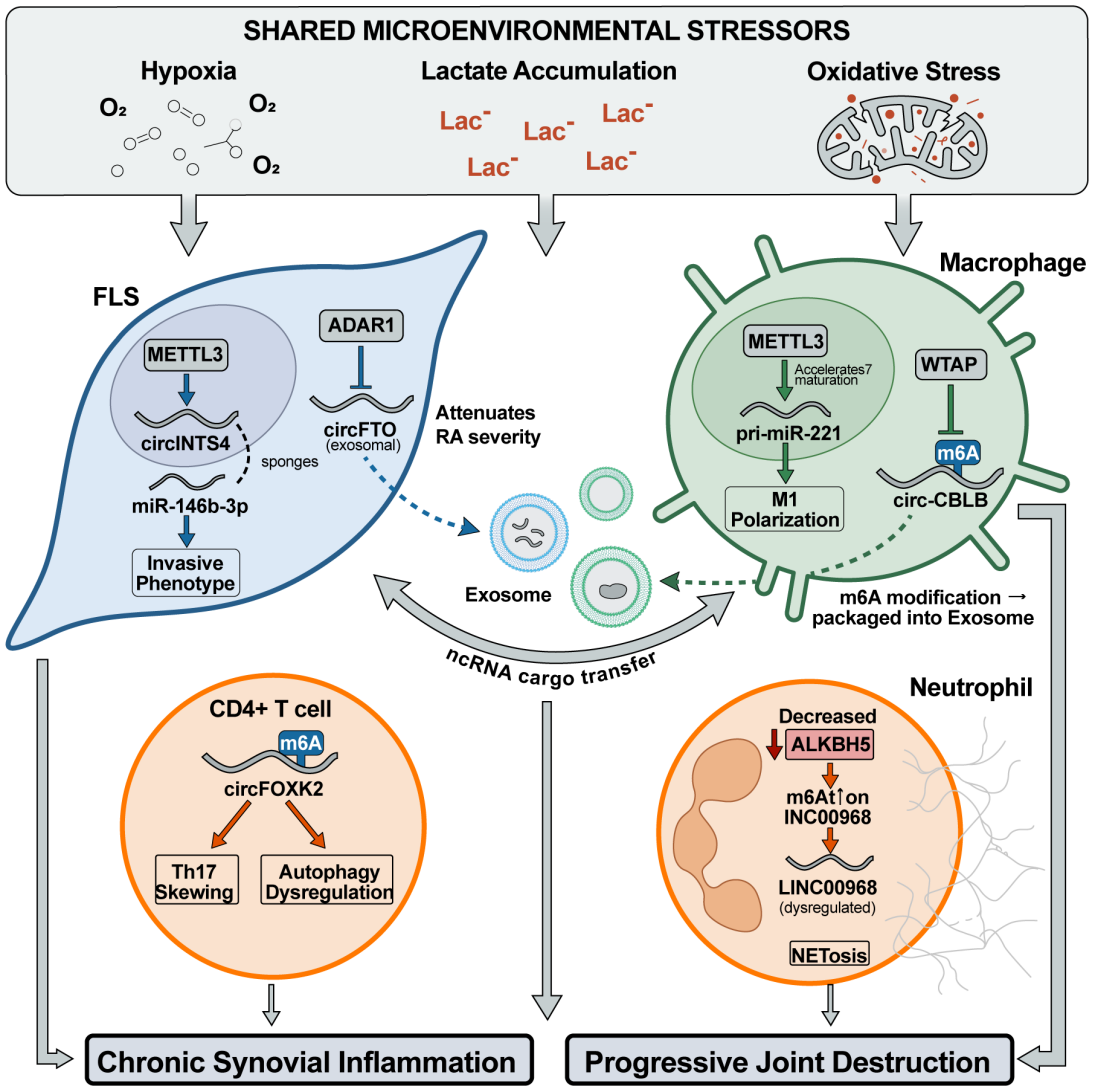
Cell-type-specific epitranscriptomic reprogramming and exosome-mediated crosstalk in the RA microenvironment. Microenvironmental perturbations shape cell-type-specific RNA methylation profiles across synovial compartments, while exosomal transfer of modified non-coding RNA cargo perpetuates intercellular inflammatory circuits. ADAR1, adenosine deaminase RNA specific 1; ALKBH5, alkB homolog 5; circRNA, circular RNA; FLS, fibroblast-like synoviocytes; lncRNA, long non-coding RNA; m6A, N6-methyladenosine; METTL3, methyltransferase-like 3; ncRNA, non-coding RNA; NETosis, neutrophil extracellular traps formation; WTAP, WT1 associated protein.

Together, these studies indicate that RNA methylation and related RNA regulatory mechanisms do not simply alter intracellular gene expression. Rather, they help define the extracellular informational landscape of the rheumatoid synovium. Through extracellular vesicles and non-coding RNA cargo transfer, pathogenic or modulatory signals can be disseminated among FLS, macrophages, T cells, neutrophils, and other synovial cell populations, thereby maintaining self-amplifying inflammatory circuits (**Figure 2**).

### 5.2 RNA methylation at the crossroads of autophagy, ferroptosis, and regulated stress responses

Pathological persistence in RA also depends on how synovial and immune cells manage cell fate decisions under chronic stress. In the inflamed synovium, cells are exposed to hypoxia, nutrient limitation, lactate accumulation, oxidative stress, mitochondrial dysfunction, and lipid peroxidation (73). Rather than undergoing exhaustion or elimination, pathogenic cells often engage adaptive stress-response programs that preserve survival and inflammatory function. RNA methylation is increasingly recognized as an important regulator of these fate decisions, operating at the intersection of autophagy, ferroptosis, and other regulated cell death pathways (74).

Autophagy provides an important example of RNA methylation-mediated stress adaptation. In neutrophils, reduced ALKBH5 expression is associated with RA disease activity and autophagic dysregulation (20). In CD4^+^ T cells, m6A-associated *circFOXK2* is linked to autophagic status and Th17 frequencies (53). These findings suggest that m6A-dependent regulation of autophagy may influence how immune cells cope with metabolic and proteotoxic stress while maintaining inflammatory activity. However, the causal relationships among RNA methylation, autophagic flux, and immune effector function remain to be further defined in cell-type-specific experimental models.

Ferroptosis has emerged as another major cell fate program shaped by RNA methylation in RA. In synovial fibroblasts, METTL3 enhances ferroptosis resistance through the m6A–IGF2BP2–*SLC7A11* axis, thereby supporting antioxidant defense and preserving the viability of pathogenic FLS under lipid peroxidation stress (31). In parallel, lactate-driven H3K18la promotes METTL1-dependent m7G modification of *NeuroD1*, providing an additional mechanism by which metabolic remodeling can reinforce ferroptosis resistance (19). These pathways suggest that m6A and m7G modifications may converge on redox and lipid stress-response programs to protect disease-driving synovial cells from elimination.

More broadly, emerging concepts such as PANoptosis emphasize that distinct forms of inflammatory programmed cell death may be interconnected rather than isolated (74). In the RA synovium, autophagy, ferroptosis, apoptosis, and NETosis may interact with one another under the influence of metabolic stress and inflammatory signaling. RNA methylation regulators may help determine whether stressed cells undergo resolution-associated death, survive as pathogenic effectors, or release inflammatory mediators that further amplify tissue damage.

Thus, RNA methylation should be viewed as a regulatory hub that links metabolic stress to cell fate plasticity. By modulating transcript stability, translation, non-coding RNA signaling, and stress-response pathways, RNA methylation can tilt the balance between inflammatory resolution and pathological persistence. (**Table 4**).

**Table 4:** RNA methylation at the interface of metabolic stress, regulated cell death, and inflammatory adaptation in rheumatoid arthritis.

Mechanistic axis	Upstream metabolic cue	RNA-level event	Pathway affected	Biological effect	Translational significance	Ref.
USP5–METTL14–m6A–GLUT1	Glycolytic escalation	GLUT1 mRNA stabilization	Metabolic adaptation	Synoviocyte survival & activation	Potential target for metabolic intervention	(27)
METTL3–m6A–IGF2BP2–SLC7A11	Oxidative and lipid stress	Antioxidant transcript stabilization	Ferroptosis resistance	Pathogenic FLS viability preservation	Candidate axis for combination therapy	(31)
H3K18la–METTL1–NeuroD1–m7G	Lactate accumulation	Chromatin-to-RNA coupling	Ferroptosis resistance	Strengthens stress tolerance	Highlights metabolite-to-RNA coupling	(19)
ALKBH5–miR-181b-5p	Inflammatory stress	miRNA maturation modulation	Apoptosis regulation	Tunes FLS survival threshold	Requires cell-specific modulation	(39)

RNA methylation acts as a regulatory hub that determines whether stressed cells undergo resolution or persist in a pathogenic state.

## 6 Clinical implications: from epitranscriptomic biomarkers to microenvironment-informed therapeutics

### 6.1 Biomarker potential: disease activity, heterogeneity, and molecular stratification

RNA methylation-associated signatures may serve as candidate high-resolution biomarkers for disease activity, molecular heterogeneity, and therapeutic responsiveness in RA. Unlike static genetic markers, epitranscriptomic features are dynamically regulated by inflammation, hypoxia, metabolic stress, and cellular activation state. However, it is crucial to emphasize that these remain preliminary experimental indicators. Unlike fully validated clinical markers (such as anti-citrullinated protein antibodies and rheumatoid factor), epitranscriptomic signatures currently lack standardized quantification assays, established clinical cut-off values, and large-scale, multi-center prospective validation. As such, their current utility is restricted to hypothesis-generating research rather than routine clinical patient stratification.

Several lines of evidence support this translational potential. Reduced ALKBH5 expression in neutrophils has been associated with clinical disease activity and impaired autophagy (20). In CD4^+^ T cells, m6A-associated *circFOXK2* correlates with Th17 frequencies and autophagic status, suggesting that RNA methylation-linked non-coding RNA signatures may reflect immune imbalance (53). In synovial fibroblasts, YTHDF2-mediated destabilization of *IL-6R* mRNA indicates that reader-associated pathways may capture inflammatory and tissue-destructive potential within the synovial microenvironment (55). Together, these findings suggest that RNA methylation regulators and their RNA targets could complement conventional biomarkers such as rheumatoid factor, anti-citrullinated protein antibodies, C-reactive protein, and erythrocyte sedimentation rate.

However, several limitations must be addressed before clinical implementation. First, RNA methylation signatures may differ substantially between peripheral blood and synovial tissue, raising questions about sample source and biological interpretability. Second, RA synovium is highly heterogeneous, with distinct inflammatory, stromal, vascular, and lymphoid niches that may harbor different epitranscriptomic states. Third, assay standardization remains a major challenge, particularly for detecting site-specific RNA modifications in clinical samples. Therefore, future biomarker studies should integrate bulk and single-cell transcriptomics, RNA modification profiling, extracellular vesicle analysis, and spatial omics to construct robust multi-marker panels (4).

A promising direction is the development of composite metabolic–epitranscriptomic signatures. Such panels could combine metabolic indicators, RNA methylation regulator expression, modified non-coding RNAs, and extracellular vesicle cargo profiles. In principle, this approach may help identify patient subgroups characterized by glycolytic FLS activation, macrophage-dominant inflammation, Th17-associated immune skewing, oxidative stress-driven NETosis, or regulated cell death resistance. These molecular subtypes could eventually support prognosis assessment and therapeutic selection, although large-scale validation in well-characterized patient cohorts will be essential.

### 6.2 Therapeutic targeting of RNA methylation enzymes: opportunities and caveats

The involvement of RNA methylation regulators in RA pathogenesis makes them attractive therapeutic candidates. In principle, targeting writers, erasers, or readers could disrupt pathogenic RNA stability, translation, non-coding RNA networks, inflammatory polarization, and resistance to regulated cell death. The reported ability of celastrol to ameliorate RA severity by targeting IGF2BP3 provides proof of concept that RNA methylation-associated reader proteins are pharmacologically tractable (32).

Nevertheless, therapeutic targeting of RNA methylation machinery must be approached cautiously. These regulators are broadly expressed and often perform essential functions in normal immune homeostasis, tissue repair, and cellular stress responses. METTL3, for example, has been implicated in ncRNA signaling, macrophage polarization, ferroptosis resistance (31, 49, 50). ALKBH5 regulates apoptosis in FLS and autophagy in neutrophils (20, 39). Moreover, YTHDF2 appears to exert protective effects in specific RA contexts by promoting *IL-6R* mRNA decay and restraining synovial inflammation (55). These examples illustrate that global inhibition of a single RNA methylation regulator may produce unintended or even paradoxical effects across different cellular compartments.

Accordingly, future therapeutic development should move beyond non-selective enzyme inhibition. More refined strategies may include cell-type-selective delivery, transcript-selective modulation, context-dependent inhibition, or targeting of specific reader–transcript interactions. For example, FLS-targeted inhibition of *METTL3*–*IGF2BP2*-dependent *SLC7A11* stabilization might sensitize pathogenic synoviocytes to ferroptotic stress, whereas preserving YTHDF2-mediated *IL-6R* decay could remain beneficial. Similarly, selective modulation of ALKBH5-dependent pathways may need to distinguish between apoptosis regulation in FLS and autophagy control in neutrophils.

Drug delivery will be a critical determinant of therapeutic feasibility. Targeted lipid nanoparticles, antibody–drug conjugate-like systems, cell-specific aptamers, engineered extracellular vesicles, or ligand-directed nanocarriers are often proposed as routes to enrich epitranscriptomic modulators in selected synovial cell populations. Nevertheless, these targeted delivery strategies remain strictly preclinical and conceptual in the context of RA. Substantial physiological hurdles—including joint-specific homing efficiency across the inflamed synovial barrier and the circumvention of off-target systemic toxicity—must be overcome. No such epitranscriptomic-directed delivery systems have entered RA clinical trials, underscoring the gap between theoretical precision and actual therapeutic feasibility.

### 6.3 Microenvironment-informed interventions: co-targeting metabolism and epitranscriptomic adaptation

A major implication of the proposed framework is that RNA methylation abnormalities should not be treated as isolated molecular defects. Since metabolic aberrations profoundly shape the epitranscriptomic landscape, then therapeutic strategies that normalize this microenvironment may destabilize pathogenic RNA methylation programs. This rationale supports the development of microenvironment-informed combination therapies.

Mechanistic evidence provides a basis for this approach. Enhanced glycolysis supports FLS activation through pathways such as the USP5–METTL14–m6A–*GLUT1* axis (27). Lactate accumulation can promote histone lactylation and METTL1-dependent m7G regulatory programs that contribute to ferroptosis resistance (19). Oxidative and lipid stress further reinforce m6A-dependent survival mechanisms, including the METTL3–IGF2BP2–*SLC7A11* axis (31). Therefore, theoretically, interventions that reduce excessive glycolytic flux or pathological lactate signaling may weaken the upstream conditions that sustain epitranscriptomic adaptation. Nevertheless, it is vital to emphasize that dual-targeting strategies (co-targeting metabolism and epitranscriptomics) remain entirely preclinical. Their proposed efficacy is currently extrapolated from *in vitro* models and related inflammatory disciplines, representing a prospective hypothesis awaiting direct proof-of-concept in clinical trials.

Such metabolic interventions should be considered complementary rather than competing strategies to current RA therapies. Conventional DMARDs, biologic agents, and targeted synthetic DMARDs remain the clinical foundation of RA management. However, combining anti-inflammatory therapy with metabolic modulation or epitranscriptomic targeting may provide an opportunity to disrupt the adaptive programs that contribute to chronicity and therapeutic resistance (74). For example, co-targeting inflammatory cytokine signaling and FLS metabolic fitness may reduce both immune activation and stromal persistence. Similarly, combining redox modulation with selective interference of ferroptosis-resistance pathways may help eliminate pathogenic synoviocytes while limiting inflammatory damage. Recent advancements in nanomedicine provide a concrete platform for these dual-targeting strategies; specifically, spatiotemporally targeted nanocapsules designed for synovial microenvironment regulation (75) and dual-prodrug nanoparticles modulating synoviocyte metabolism (76) have shown profound preclinical efficacy in suppressing chronic joint destruction.

Importantly, microenvironment-informed therapy must be guided by careful patient stratification. Not all patients with RA are likely to exhibit the same dominant metabolic or epitranscriptomic phenotype. Some may be characterized by highly glycolytic FLS-rich synovitis, whereas others may show macrophage-dominant inflammatory programs, lymphoid aggregates, or neutrophil-driven oxidative injury. Composite biomarkers integrating metabolic status, RNA methylation signatures, cellular composition, extracellular vesicle cargo, and broader epigenetic features may therefore be necessary to identify patients most likely to benefit from specific combination strategies (20, 25).

Overall, the conceptual therapeutic value of the metabolic–epitranscriptomic axis may lie in rationally designed combinations that target both microenvironmental perturbations and downstream RNA-dependent adaptive mechanisms. Such strategies theoretically could help shift the RA synovium from a state of persistent pathogenic adaptation toward inflammatory resolution. However, it must be explicitly stated that co-targeting metabolism and epitranscriptomics is currently a preclinical hypothesis. Its proposed efficacy is extrapolated from *in vitro* models and oncology, and such combinatorial interventions require rigorous toxicological and efficacy evaluations *in vivo* before they can be considered true clinical alternatives. (**Tables 5**, **6**).

**Table 5:** Evidence-based translational implications of the metabolic-epitranscriptomic axis.

Translational domain	Candidate markers/targets	Evidence status	Clinical utility	Ref.
Disease activity monitoring	ALKBH5, YTHDF2, circFOXK2-associated signatures	Direct RA evidence (Patient samples/Moderate)	Reflects local inflammatory activity and cellular stress states	(20, 53, 55)
Molecular stratification	m6A/m7G regulator expression profiles; ncRNA signatures	Indirect translational evidence (Metabolomic and peripheral blood profiling)	Identifies biologically distinct patient subsets	(20, 25)
Therapeutic targeting	METTL3, METTL1, ALKBH5, IGF2BP3	Preclinical validation (In vivo animal models)	Offers mechanistically defined intervention points	(32)
Combination therapy	Metabolic modulation plus epitranscriptomic targeting	Inferred/Preclinical concept	May disrupt upstream drivers of disease persistence	(74–76)

This table summarizes domains where direct observational or preclinical *in vivo* evidence within RA models supports the translational utility of RNA modification machinery.

**Table 6:** Hypothesis-generating frameworks for precision medicine in RA.

Precision Medicine Framework	Proposed Integration	Clinical Rationale	Main Challenge	Ref.
Composite Signatures	Integrating metabolic status with m6A-profiles and EV cargo	Supports individualized prognosis and highly selective treatment prediction	Requires large-scale validation and robust multi-omics cohorts	(20, 25)
Subtype Stratification	Autoantibody serostatus (e.g., ACPA+/-) combined with metabolic-m6A profiling	Tailoring systemic (ACPA+) vs. localized (ACPA-) dual-targeted therapies	Defining distinct metabolic-epitranscriptomic signatures between subgroups	(77)
Stage-Specific Windows	Targeting acute metabolic stress (early) vs. chronic epitranscriptomic memory (late)	“Window of opportunity” intervention vs. reversing entrenched joint chronicity	Dynamic temporal evolution makes precise therapeutic timing difficult	(78, 79)

This table outlines conceptual strategies that rely on extrapolating the metabolic-epitranscriptomic axis into precision medicine. These frameworks represent the next frontier in therapeutics but require rigorous prospective validation.

*ACPA, anti-citrullinated protein antibodies.*

### 6.4 Precision medicine framework: tailoring strategies to RA subtypes and stages

While jointly targeting metabolic stress and RNA methylation represents an intriguing conceptual frontier, it is vital to acknowledge that patient stratification based on these specific pathways is entirely theoretical at present. However, looking toward future translational efforts, maximizing potential clinical efficacy would likely require a paradigm shift towards precision medicine. Because RA encompasses profound clinical heterogeneity, deploying any future dual-targeting therapies would theoretically need to be rigorously stratified according to distinct disease subtypes and temporal stages.

#### 6.4.1 Stratification by RA subtypes (ACPA-positive vs. ACPA-negative)

RA is fundamentally classified into distinct pathological entities based on autoantibody serostatus, particularly anti-citrullinated protein antibodies (ACPA). As highlighted in classic immunological paradigms, ACPA-positive and ACPA-negative RA possess fundamentally divergent underlying pathomechanisms and genetic backgrounds (77).

ACPA-positive RA is generally characterized by a more aggressive disease trajectory, higher systemic inflammatory burden, and more severe bone erosion. In these patients, the overall metabolic-epitranscriptomic network is likely skewed toward robust systemic autoimmune amplification. Consequently, clinical strategies for this subtype should prioritize aggressive, dual-targeted inhibitors that systemically uncouple the metabolic-m6A pathways driving severe autoimmunity and structural destruction.

Conversely, ACPA-negative RA typically exhibits distinct etiopathogenesis and may involve different modes of metabolic rewiring that sustain joint inflammation. Therapeutic interventions for ACPA-negative cohorts should therefore be tailored to target the specific metabolic and epitranscriptomic signatures that drive localized synovial inflammation, avoiding the over-suppression of pathways primarily linked to classic ACPA production.

#### 6.4.2 Stage-specific therapeutic windows (early vs. late RA)

The metabolic and epitranscriptomic landscape within the rheumatoid joint is not static but evolves dynamically, necessitating stage-specific therapeutic windows.

In early-stage RA, the pathology is characterized by acute metabolic shifts (such as rapid glycolytic surges and aberrant metabolite accumulation) that initiate the inflammatory cascade (78). Interventions during this critical “window of opportunity” should aim to deploy metabolism-RNA methylation modulators to promptly reverse these acute epigenetic imprints, thereby breaking the cycle of inflammation before it progresses to chronicity.

In late-stage or refractory RA, the local joint microenvironment transitions into a state of chronic metabolic exhaustion, severe hypoxia, and established epitranscriptomic “memory” (79). In this advanced phase, the therapeutic priority shifts from merely suppressing early inflammation to preventing irreversible joint remodeling and pannus-driven destruction. Consequently, treatments for late-stage RA must focus on dismantling the deeply entrenched metabolism-m6A feedback loops that sustain tissue chronicity and drug resistance, utilizing targeted delivery systems to remodel the hostile microenvironment within the destroyed joints.

## 7 Conclusion and future perspectives

Rheumatoid arthritis is increasingly recognized as a disease sustained by persistent adaptation to a metabolically hostile synovial microenvironment (80). Current evidence indicates that this metabolic–epitranscriptomic coupling operates across multiple cellular compartments. In fibroblast-like synoviocytes, RNA methylation supports glycolytic fitness, invasive behavior, and resistance to cell death, while in immune cells, it contributes to inflammatory polarization, Th17 skewing, and excessive NETosis. Crucially, RNA methylation regulators are context-dependent; their biological effects depend on the modified transcript, the reader protein, the cellular lineage, and the local metabolic state.

Moving forward, translating this knowledge into clinical practice requires addressing several key unresolved questions. First, the spatial organization of RNA methylation within the inflamed joint remains an enigma; future studies must leverage spatial epitranscriptomics to map these networks across distinct synovial microdomains. Second, the causality between metabolic stress and writer/eraser/reader activity requires precise deconvolution to clarify whether metabolic shifts exclusively initiate RNA modification, or if epitranscriptomic rewiring primarily drives the metabolic collapse. Third, assay standardization is mandatory before epitranscriptomic profiles (including non-m6A marks such as m7G and ac4C, non-coding RNAs, and extracellular vesicle cargo) can transition from experimental findings to reliable clinical diagnostics. Finally, achieving therapeutic selectivity is paramount. Because RNA methylation machinery is fundamental to normal physiology, future strategies must prioritize cell-selective and microenvironment-informed approaches—such as nanotechnology-enabled targeted delivery—to disrupt these self-reinforcing loops without inducing systemic toxicity (81, 82). Ultimately, resolving these challenges will improve patient stratification and guide precision-oriented combination therapies in RA.
